# A combinatorial native MS and LC-MS/MS approach reveals high intrinsic phosphorylation of human Tau but minimal levels of other key modifications

**DOI:** 10.1074/jbc.RA120.015882

**Published:** 2021-01-13

**Authors:** Friedel Drepper, Jacek Biernat, Senthilvelrajan Kaniyappan, Helmut E. Meyer, Eva Maria Mandelkow, Bettina Warscheid, Eckhard Mandelkow

**Affiliations:** 1 Group of Biochemistry and Functional Proteomics, Institute of Biology II, University of Freiburg, Freiburg, Germany; 2 Signalling Research Centres BIOSS and CIBSS, University of Freiburg, Freiburg, Germany; 3 DZNE (German Center for Neurodegenerative Diseases), Bonn, Germany; 4 Department of Neurodegenerative Diseases and Geriatric Psychiatry, University of Bonn, Bonn, Germany; 5 Medical Proteome Center, Ruhr-University Bochum, Bochum, Germany; 6 Department of Biomedical Research, Leibniz-Institute for Analytical Sciences (ISAS), Dortmund, Germany; 7 CAESAR Research Center, Bonn, Germany

**Keywords:** Tau protein (Tau), mass spectrometry (MS), phosphorylation, Alzheimer's disease, protein aggregation, LC-MS, native mass spectrometry

## Abstract

Abnormal changes of neuronal Tau protein, such as phosphorylation and aggregation, are considered hallmarks of cognitive deficits in Alzheimer's disease. Abnormal phosphorylation is thought to precede aggregation and therefore to promote aggregation, but the nature and extent of phosphorylation remain ill-defined. Tau contains ∼85 potential phosphorylation sites, which can be phosphorylated by various kinases because the unfolded structure of Tau makes them accessible. However, methodological limitations (*e.g.* in MS of phosphopeptides, or antibodies against phosphoepitopes) led to conflicting results regarding the extent of Tau phosphorylation in cells. Here we present results from a new approach based on native MS of intact Tau expressed in eukaryotic cells (Sf9). The extent of phosphorylation is heterogeneous, up to ∼20 phosphates per molecule distributed over 51 sites. The medium phosphorylated fraction P_m_ showed overall occupancies of ∼8 P_i_ (± 5) with a bell-shaped distribution; the highly phosphorylated fraction P_h_ had 14 P_i_ (± 6). The distribution of sites was highly asymmetric (with 71% of all P-sites in the C-terminal half of Tau). All sites were on Ser or Thr residues, but none were on Tyr. Other known posttranslational modifications were near or below our detection limit (*e.g.* acetylation, ubiquitination). These findings suggest that normal cellular Tau shows a remarkably high extent of phosphorylation, whereas other modifications are nearly absent. This implies that abnormal phosphorylations at certain sites may not affect the extent of phosphorylation significantly and do not represent hyperphosphorylation. By implication, the pathological aggregation of Tau is not likely a consequence of high phosphorylation.

Tau (*MAPT*, Uniprot P10636) is a developmentally regulated protein promoting microtubule-based functions like axonal transport in neurons. The properties of Tau can be altered by various posttranslational modifications (PTMs), most conspicuously by phosphorylation (1). The aggregation of Tau into amyloid-like insoluble filaments is one of the hallmarks of neurodegenerative diseases called tauopathies, including Alzheimer's disease (AD). Early observations suggested a high level of phosphorylation of Tau preceding that of aggregation (2, 3), which was therefore interpreted to promote aggregation and is used as an early marker of neuronal degeneration. Tau-targeted therapies, aiming at reducing Tau levels, Tau distribution, or Tau modifications, have emerged as potential strategies for treating tauopathy in patients (4, 5, 6). Likewise, quantitative diagnostics, including the analysis of soluble Tau from cerebrospinal fluid, is gaining importance (7). These examples underscore the need for improved quantitative diagnostics.

Initial quantitative assessments of Tau-bound phosphate yielded ∼8 P_i_ per Tau molecule in AD-Tau (8, 9). This was compared with fetal Tau at ∼7 P_i_ per Tau (10) and adult human cytosolic Tau at ∼2 P_i_ (8, 9), interpreted to mean that the extent of Tau phosphorylation is abnormally high in AD. In parallel, phosphorylation-dependent antibodies and phosphopeptide mapping approaches were developed to identify phosphoepitopes on Tau (*e.g.* (11); for a current list see RRID:SCR_013601). However, these antibody-based methods were not well-suited for the determination of the occupancy of the P-sites and the overall state of phosphorylation.

The advent of Edman degradation and MS resulted in increased sensitivity and specificity of detection of PTMs on Tau protein. In particular, the combination of HPLC with tandem MS (HPLC-MS/MS) resulted in enhanced coverage of the Tau sequence with newly identified P-sites (12, 13).

However, to address the issue of abnormal phosphorylation *versus* aggregation, an experimental system was needed which enabled high phosphorylation of defined Tau isoforms. This was achieved with the expression of full-length human Tau (2N4R) in transfected Sf9 cells, which yields high protein levels (up to 230 µm in cells) and high diversity of P-sites, as judged by antibody reactivity and MALDI-TOF MS analysis (14). Depending on cell treatment (without or with phosphatase inhibitor okadaic acid), the overall occupancy was estimated around 12 P_i_ or 20 P_i_. Hence the fractions were termed P12 or P20, renamed in this paper to P_m_ and P_h_ to indicate medium *versus* high phosphorylation state, and in contrast to unphosphorylated P_o_-Tau expressed in *Escherichia coli* (Fig. 1, *A*–*C*). None of the phosphorylated Tau proteins showed an enhanced tendency for aggregation (14).

**Figure 1:**
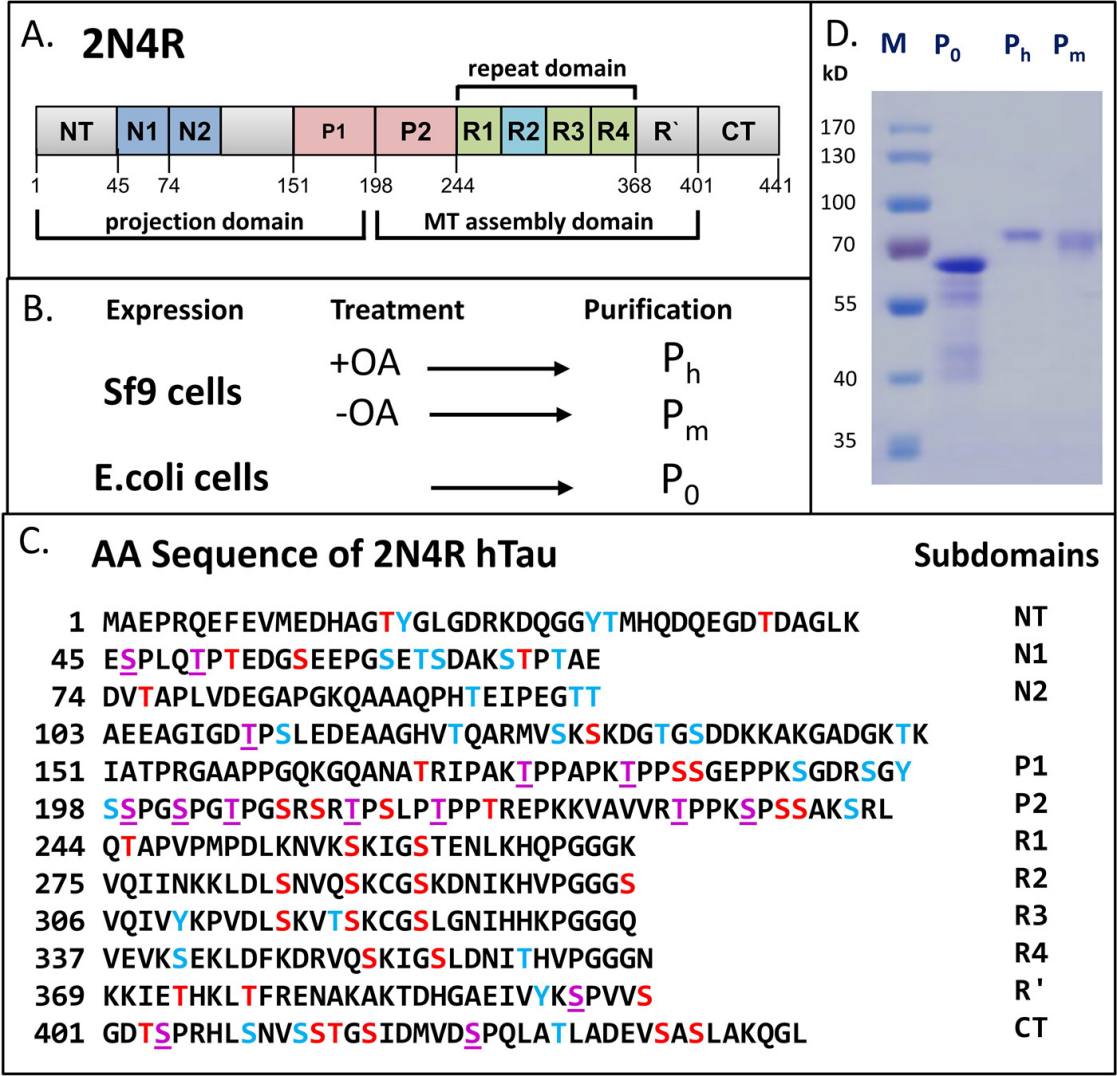
**Overview: Phosphorylation of hTau-2N4R expressed in Sf9 cells and *E. coli*.***A*, diagram of domains of hTau40 (2N4R or Tau-F, Uniprot ID P10636-8), the largest human isoform of CNS Tau consisting of 441 residues with the three alternatively spliced inserts N1, N2, and R2. The N-terminal half represents the projection domain, and the C-terminal half contains the four pseudo-repeats (R1–R4) which, in combination with their flanking domains P2 and R′, represent the microtubule assembly domain. *B*, schematic representation of production of unphosphorylated Tau (P_0_) in *E. coli* (prokaryote) and hyperphosphorylated Tau (P_m_ and P_h_) in Sf9 cells (eukaryote). Okadaic acid (*OA*) treatment increases the phosphorylation of Tau which yields P_h_-Tau. *C*, amino acid sequence of Tau (2N4R, 441 residues) showing phosphorylation sites identified in this study (*red and purple letters*; *purple = Pro directed)*) and further potential phosphorylation sites (not detected so far, *blue letters*) (total of 85 sites, 45 Ser, 35 Thr, and 5 Tyr). Note that only a minority of phosphorylation sites of 37% was detected in the N-terminal part of the sequence (up to residue ∼150, compared with 71% in the C-terminal part of the protein). Note also that none of the five Tyr residues was phosphorylated. *D*, SDS-PAGE analysis stained with Coomassie Blue showing P_0_ Tau purified from *E. coli* and P_m_- and P_h_-Tau purified from Sf9 cells. Note the upward shift in *M_r_* value with increasing phosphorylation, from 55 kDa (P_0_-Tau) to 68 kDa for P_h_-Tau (compared with the theoretical molecular mass values of ∼45,850 Da and ∼46,863 Da). This shift is characteristic for AD-Tau.

Nonetheless, the same fractions were studied by a novel MS-based assay, FLEXITau (15), designed to determine the locations and occupancies of all P-sites quantitatively by comparison with isotopically labeled peptide forms. This procedure yielded overall occupancies of only 7 and 8 P_i_ per Tau molecule in P_m_-Tau and P_h_-Tau, respectively, with a broad spread (± 5 around the mean) and up to 23 observed P-sites. Other MS approaches, designed to identify P-sites in Tau from AD brains and cerebrospinal fluid, revealed ∼30 P-sites but not the overall occupancies (16).

In the present work, we employed native MS to determine the extent of Tau phosphorylation in cells and to clarify the variations between different methods. MS of intact proteins, such as top-down MS (17) and native MS (18, 19), allow quantification of different proteoforms without requiring prior proteolytic cleavage into peptide fragments or comparison with reference standards (20). The procedure revealed that full-length Tau in the P_m_ fraction contained 8 ± 5 P_i_ and in the P_h_ fraction 14 ± 6 P_i_. Subsequent analysis of phosphorylated peptides revealed up to 51 P-sites in Tau, with variable site occupancies. Finally, the method sensitively detected a low level of different cellular proteins associated with Tau.

## Results

### Analysis of intact full-length phospho-Tau by native MS

**Figure 2:**
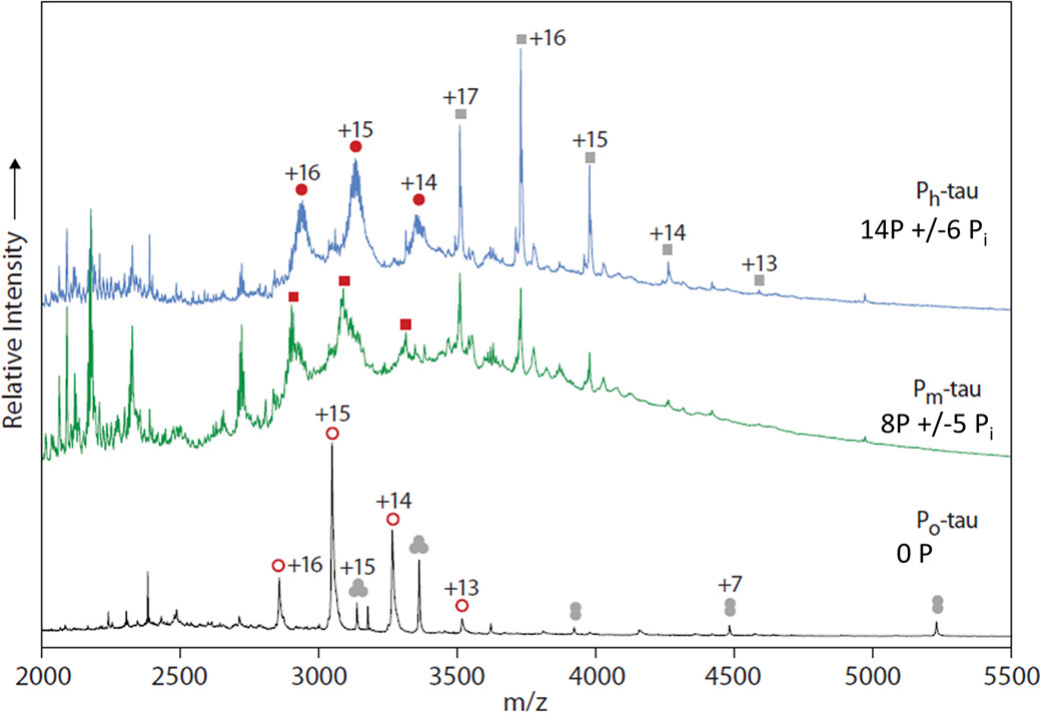
**Analysis of hTau-2N4R expressed in Sf9 cells by native MS.** 20 µm of purified proteins in 200 mm ammonium acetate, pH 7.6, were analyzed by nanoflow electrospray ionization quadrupole TOF MS. Representative mass spectra for the highly phosphorylated states P_h_-Tau (*top*) and P_m_-Tau (*middle*) and unphosphorylated. P_0_-Tau (*bottom*) are shown. *Top*, main signals were assigned to charge state series of P_h_-Tau (molecular mass of 46,883 Da; *filled red circles*) and a copurified component with a molecular mass of 59,642 Da (*gray squares*). *Middle*, spectrum of P_m_-Tau displaying peaks assigned to a series of equivalent charge states but shifted toward lower *m*/*z* compared with those for P_h_-Tau (red squares). Further signals match closely with those in the spectrum of P_h_-Tau. *Bottom*, spectrum of control Tau P_0_ consisting of a series of charge states indicating a molecular mass of 45,724 Da (*open red circles*). A further series was assigned to a molecular mass of 47,085 Da. It was attributed to a trimeric species (*gray circles*) because it decomposes upon increasing collisional activation into highly charged (centered at +9, below *m*/*z* 2,000) monomeric and charge-stripped dimeric species (charges +6 to +8). Its monomeric mass of 15,691 Da matches the theoretical mass of the *E. coli* chaperone protein *skp* (see Table S4), taking into account removal of its N-terminal 20–amino acid signal peptide.

**Figure 3:**
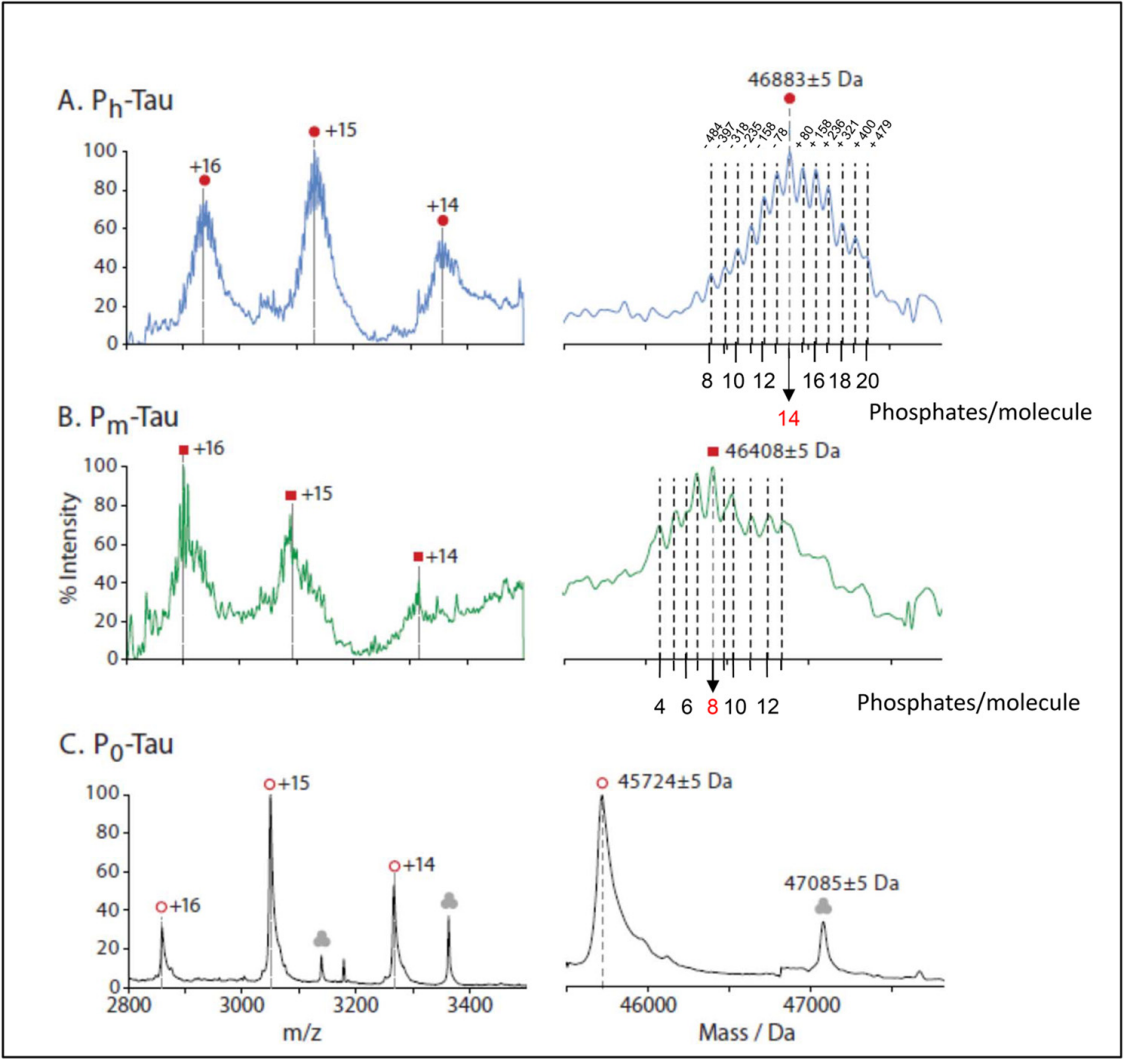
**Phosphorylation of hTau-2N4R expressed in Sf9 cells analyzed by native MS.***Left*, signals in the range between 2800 and 3500 *m*/*z* attributed to Tau proteins. *Right*, corresponding charge state–deconvoluted spectra. *A*, the mass spectrum of P_h_-Tau (*top*) displays a center mass of 46,883 Da (*filled red circles*) and additionally six peaks on both sides equally spaced by ∼80 Da. *B*, P_m_-Tau (*middle*) displays peaks of similar width shifted toward lower *m*/*z* compared with those for P_h_-Tau, which can be assigned to the equivalent charge states and indicate a molecular mass of ∼46,408 Da (*red squares*). *C*, the spectrum of control Tau P_0_ consists of a series of charge states resulting in a comparatively sharp peak at a mass of 45,724 Da (45,850 −131Da ± 5 because of cleavage of first residue Met-1; *open red circles*). A further series was assigned to a molecular mass of 47,085 Da and was attributed to a trimeric species (*gray circles*) because it decomposes upon increasing collisional activation into highly charged (centered at +9, below *m*/*z* 2000) monomeric and charge-stripped dimeric species (charges +6 to +8). Its monomeric mass of 15,691 Da matches the theoretical mass of the *E. coli* chaperone protein *skp* (see Table S4), taking into account removal of its N-terminal 20–amino acid signal peptide.

Analysis of tryptic peptides by HPLC-MS/MS reveals sites of protein phosphorylation but does not directly monitor the extent of phosphorylation per molecule (*i.e.* overall occupancy) because the detection efficiency varies between phosphorylated and nonphosphorylated peptides. This problem is circumvented by native MS of full-length Tau (18). We compared the phosphorylation status of two differently phosphorylated Tau fractions expressed in eukaryotic Sf9 cells with unphosphorylated Tau from *E. coli* bacteria (Fig. 1*B*). Fig. 2 shows native MS spectra of unphosphorylated control Tau (termed P_o_, expressed in *E. coli*, *bottom*), Tau from the fraction P_m_-Tau (medium level phosphorylation, expressed in Sf9 cells, *middle*), and highly phosphorylated fraction P_h_-Tau (in presence of phosphatase inhibitor okadaic acid, *top*). For P_o_-Tau, the main signals were recorded between *m*/*z* 2500 and 3500. By charge state deconvolution, a series of signals corresponding to charges +16 to +13 can be related to a molecular mass of 45,724 Da (Fig. 2, *open red circles*; see also Fig. 3). Native MS spectra of P_m_-Tau and P_h_-Tau show several matching signals in the range below *m*/*z* 2750 and above *m*/*z* 3500 (Fig. 2, *middle and top spectrum*). In between, broad peaks are visible for both P_m_-Tau and P_h_-Tau which can be assigned to a series of charge states +16 to +14 (*red squares* for P_m_-Tau and *filled red circles* for P_h_-Tau). Both series are shifted toward higher *m*/*z* compared with the corresponding charge states of the control Tau P_o_. Further peak series in P_o_-Tau were attributed to a trimeric complex (*gray circles*, *bottom trace*) of the *E. coli* periplasmic chaperone protein Skp (molecular mass of full-length Skp 17,688 Da, reduced to 15,691 Da after cleavage of 20-residue signal peptide (see Table S4)) and to an unknown protein of 59,642 ± 5 Da (*gray squares*, *top and middle traces*) copurified with P_m_-Tau and P_h_-Tau from Sf9 cells.

Native MS spectra in the range of *m*/*z* 2800 to 3500 (Fig. 3, *left*) were analyzed by charge state deconvolution to generate zero-charge mass spectra revealing further differences between these Tau protein forms (Fig. 3, *right*). For P_o_-Tau from *E. coli* (*bottom*), a sharp peak indicates a molecular mass of 45,724, Da which is 126 Da lower than the molecular mass of unmodified full-length Tau (2N4R, calculated at 45,850 Da). Taking into account the mass accuracy of ±5 Da, this can be explained by a cleavage of the N-terminal methionine (131 Da; Fig. 3). For P_h_-Tau from Sf9 cells (Fig. 3, *top*), a broader peak in the zero-charge mass spectrum displays a center mass of 46,883 Da and up to six additional peaks on both sides equally spaced by ∼80 Da. The center mass would correspond to an N-terminally acetylated htau40 carrying 14 ± 6 P_i_ groups.

The spectrum of P_m_-Tau was less well-resolved and possibly disturbed by a feature at *m*/*z* 2750, which is present in the P_h_-Tau spectrum at much lower intensity. A charge state deconvolution results in a center mass of 46,408 ± 400 Da, indicating 8 ± 5 P_i_ (Fig. 3, *middle row*). Of note, although the native MS spectrum of P_m_-Tau was less well-defined, all three charge states of +16 to +14 are clearly shifted toward lower *m*/*z* values compared with the P_h_-Tau spectrum but remained at higher *m*/*z* values compared with the corresponding charge states in the control spectrum of P_0_-Tau (see Figure 2, Figure 3).

### Identification of peptide sequences and posttranslational modifications by LC–MS/MS

#### Phosphorylation

For protein identification and detection of PTMs, the protein samples were subjected to reduction of cysteine residues, alkylation of free thiol groups, and digestion with trypsin, followed by LC–MS/MS analysis. The identified tryptic peptides covered 95% of the Tau sequence and allowed us to detect sites of posttranslational modifications of the Tau proteins. We therefore analyzed our LC–MS/MS data for the following amino acid modifications: acetylation of the protein N terminus, phosphorylation of serine, threonine, or tyrosine, acetylation of lysine, and ubiquitination of lysine. For purified P_m_-Tau and P_h_-Tau, only N-terminally acetylated forms of peptides containing the protein N terminus (without Met1) were detected. In contrast, for control P_0_-Tau, these two peptides were identified in their nonmodified, nonacetylated N-terminal peptide form. We therefore deduced from the protein sequence (residues 2–441) average masses of 45,718.2 Da for the nonN-terminally acetylated *E. coli* Tau (P_0_) and 45,760.2 Da for the N-terminally acetylated form of htau40 (P_m_ and P_h_) in the absence of further modifications.

The total number of P-sites on serine and threonine residues on Tau identified at a localization probability higher than 0.75 was 51 sites (32 Ser, 19 Thr). No phospho-tyrosines were detected (for P-sites see Table S1; for procedures to derive site probabilities see “Experimental procedures”). No P-sites were detected for *E. coli* P_0_-Tau, as expected. The P-site analysis of Tau from Sf9 cells confirmed the majority of P-sites found in previous studies (14, 15), including all prominent Alzheimer-specific antibody phosphoepitopes, notably those that require pairs of phosphorylated residues (*e.g.* antibodies AT-8, PHF-1, and AT100). Furthermore, the analysis revealed 10 additional P-sites (Thr-373, Thr-377, Ser-400, Thr-403, Ser-413, Thr-414, Ser-416, Ser-422, Ser-433, and Ser-435; see Table S1) in the C-terminal domain of Tau, which have been reported for Tau from human AD brain (12, 13) but were absent in our previous studies. Interestingly, six P-sites (Thr-245, Ser-285, Ser-293, Ser-316, Ser-320, and Ser-352; see Table S2) were identified in the repeat domain which are not annotated in the collection of P-sites from AD brains (12, 13).

The highly phosphorylated fraction P_h_ contains 14 Pi (± 6 Pi) per Tau molecule, indicating a maximal value of 20 Pi (higher values are possible but are not resolved in the spectrum). The heterogenous P-site distribution over the entire length of the Tau chain is generally in line with previous findings (15) (and see “Discussion”).

#### Other posttranslational modifications

In contrast to the many identified P-sites, it is remarkable that other well-documented PTMs of Tau reported for human and mouse brain are close or below the detection threshold in Tau from Sf9 cells. This includes lysine acetylation and lysine ubiquitination, both of which have been linked to neurodegeneration in AD (21, 22, 23) and *O*-GlcNAc modification of serine and threonine, which was reported to prevent phosphorylation and aggregation of Tau (24, 25). Of note, in contrast to the high number of detected P-sites and their high abundancies, other PTMs, if significant at all, were observed only sporadically and in low intensity. Further validation and characterization of such low abundant additional modifications would need specialized approaches and is beyond the scope of the present study.

#### Tau from neuronal origin

In addition to Tau expressed in cell models, we studied the phosphorylation status of Tau isolated from brains of transgenic mice, using LC–MS/MS and native MS methods. The LC–MS/MS analysis was performed on neuronal Tau from transgenic mice expressing the mutation Tau^A152T^ (a risk factor for progressive supranuclear palsy (26)) purified by immunoprecipitation using the Tau^A152T^ specific antibody 1C5. The analysis showed that human Tau^A152T^ expressed in mice is highly phosphorylated at five sites (Thr-181, Thr-231, Ser-199, Ser-202, and Ser-396; P-site probability > 0.75). These sites are consistent with the subset of P-sites in Sf9 cells with the highest occupancy (>50%) (15) and with prominent P-sites of Tau in AD brain. However, native MS did not reveal a specific spectrum of Tau from neuronal origin, mainly because of the inherent heterogeneity of neuronal Tau proteins, low concentration, and contamination by other proteins. This problem will require further development.

### Proteins copurifying with Tau identified by LC–MS/MS

The high sensitivity of LC–MS/MS revealed the presence of tryptic peptides originating from traces of other proteins accompanying Tau through the purification procedure. The recombinant Tau proteins (P_0_ from *E. coli*, P_m_ and P_h_-Tau from Sf9 cells) were purified by making use of the heat stability of Tau protein whereby nearly all other proteins are denatured and precipitated, whereas Tau remains in solution because of its hydrophilic nature. The detected small amounts of cellular Tau-binding proteins also passed through the boiling step and were still present in gel filtration column fractions containing the separated Tau proteins. One possibility to explain the presence of these proteins is their high affinity for Tau protein. Thus, the experiments yielded a list of Tau-interacting proteins that might become interesting for analyzing possible Tau interactions in other cell types (see Table S4 for P_0_-Tau from *E. coli* and Table S5 for P_m_-Tau and P_h_-Tau from Sf9 cells). A substantial part of identified proteins are known for their roles in interacting with RNA or DNA and with chaperones (see “Discussion”).

## Discussion

### Significance of Tau phosphorylation in AD research

Tau is a neuronal protein whose best-known role is to stabilize microtubules in axons, thus supporting their role as tracks for axonal transport and as struts of axonal shape (27). Tau has a hydrophilic character, contains many charged residues, is highly soluble, and has a mostly unfolded structure; as such it can interact with many cellular components in addition to microtubules (1). The interactions can be modulated by various PTMs, notably by phosphorylation at Ser, Thr, or Tyr residues (up to 85 potential sites). It has been reported that in physiological conditions human fetal Tau is highly phosphorylated (∼7–10 Pi per Tau molecule), compared with human adult Tau (∼2 Pi), and high again for human AD patients (7–10 Pi) (8, 9, 10). However, these values need to be reinterpreted in the light of later discoveries (see below). In hibernating animals, Tau becomes highly phosphorylated in a reversible manner, corresponding to a cyclic regression and reappearance of dendritic trees, which indicates that Tau phosphorylation may play a role for neuronal plasticity (28). Many cell and animal models of Tau have been developed to study its functions. In particular, Sf9 cells served as early examples of Tau's role in the control of process outgrowth via microtubules and actin filaments (29, 30) and the importance of reversible phosphorylation (31). Beyond cell biological issues, the major interest in Tau arises from its property as a hallmark of brain diseases, notably Alzheimer's and other tauopathies (32). The finding that Tau becomes aggregated into amyloid filaments in AD and appears hyperphosphorylated triggered an extended search for kinases and phosphatases responsible for Tau's pathological state (33, 34), searches for P-sites on Tau (13), and treatments based on these results (35). The aim was to identify P-sites as early indicators of pathology in brain tissue, cerebrospinal fluid, or blood (36, 37), and as indicators of vulnerable brain circuits affected during Braak stages (38). Despite this progress, the relationship between Tau's phosphorylation and aggregation remains enigmatic in view of its exceptional solubility.

### Methods development

In the past, the main tools for identifying phosphorylation sites of proteins were based on tryptic cleavage, combined either with TLC of ^32^P-labeled phosphopeptides, peptide sequencing (39), or MS of peptides (*e.g.* MALDI-TOF MS) (40). For the case of Tau, these methods yielded information on P-sites involved in AD (14, 41, 42). A limitation common to these methods was that the extent of phosphorylation of peptides or the whole protein remained uncertain. For peptides, this was partially overcome by using isotope-labeled standards (15, 43). However, the correlation between P-sites and the global extent of phosphorylation remained a problem. The early studies of global phospho-occupancy, obtained by ashing and color reaction of samples, revealed average global occupancies but carried no information on occupancy of individual P-sites. Yet, knowledge of both the degree of phosphorylation and the main P-sites would be important for assessing the cellular functions (*e.g.* control of microtubule assembly, phase separation in the cytoplasm) and the pathological state of Tau (*e.g.* somatodendritic missorting, pathological aggregation). This can be achieved by combining native MS with LC–MS/MS, as described here. We will restrict our discussion to some issues that have received attention in the Tau field during recent years.

### Overall extent of phosphorylation

In previous work (14), we described three major fractions of phospho-Tau, termed P0, P12, and P20, whose degree of phosphorylation was estimated from the peaks observed by MALDI-TOF MS. Fraction P0 corresponded to the unphosphorylated protein expressed in *E. coli*. Fraction P12 was derived from Sf9 cells after expression of full-length Tau (2N4R), whose mass was consistent with additional ∼12 Pi. Fraction P20 was also from Sf9 cells, with phosphatase inhibitor okadaic acid added during preparation, consistent with additional ∼20 Pi.

**Figure 4:**
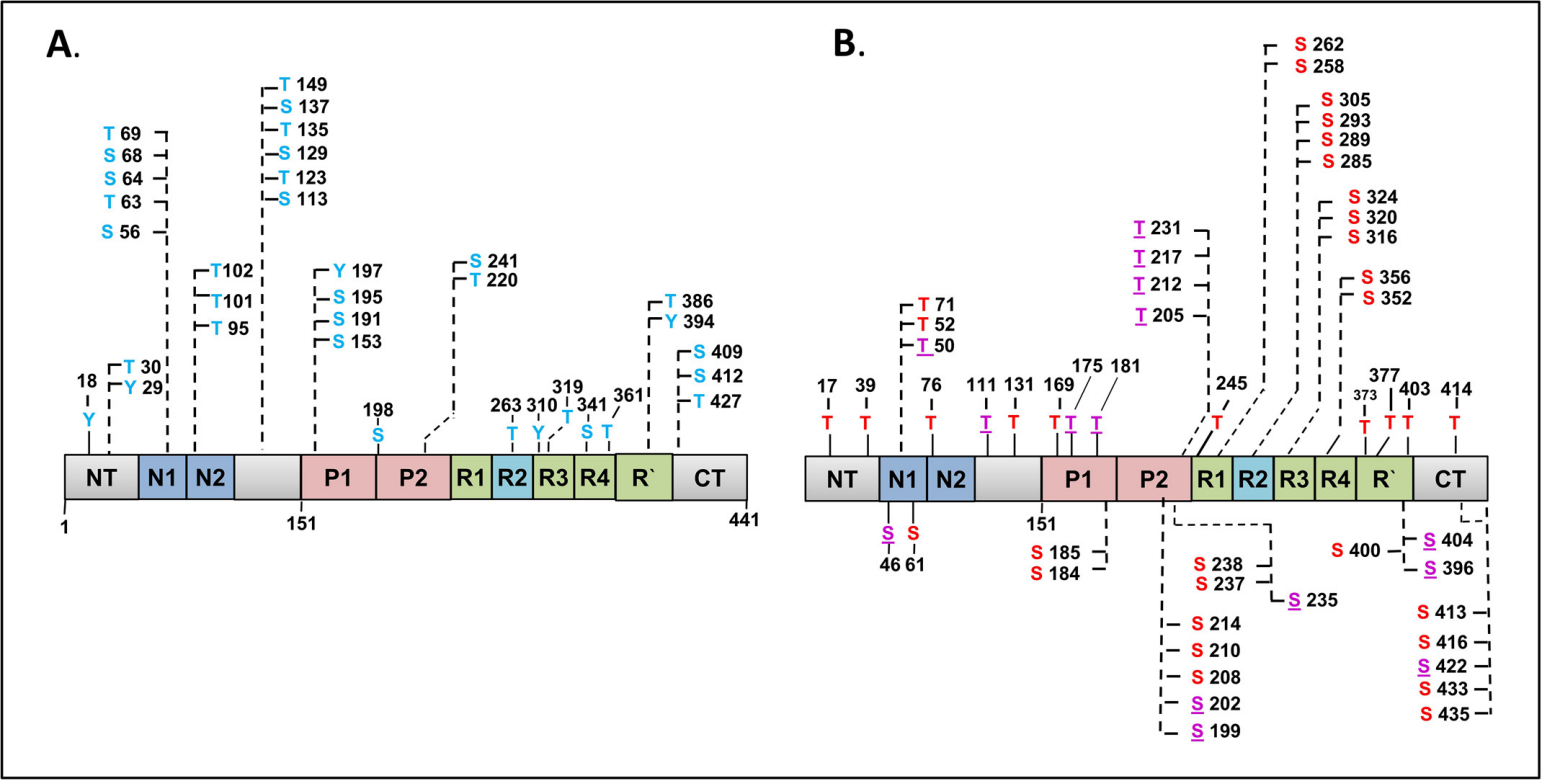
Bar diagram of full-length human Tau-2N4R protein with distribution of nonobserved *versus* observed phosphorylation sites in Sf9 cells. Thr residues shown above bar, Ser residues below. *A*, nonphosphorylated sites are located mainly in the N-terminal half (*blue*). *B*, observed phosphorylation sites are located mainly in the C-terminal half (*red*), especially in SP or TP motifs (*purple underlined*).

The same types of fractions were studied by Steen and co-workers (15) using the FLEXITau method where quantification was done with reference to isotope-labeled full-length Tau. The fractions were denoted as P-Tau (equivalent to the P12 preparation of (14)), but with a lower overall occupancy of 7 P_i_, and PP-Tau (equivalent to P20 preparation), with an overall occupancy of 8 P_i_. In addition, the method revealed the individual occupancies of 17 sites, including those of the major Tau antibodies (Figure 3, Figure 4 in (15)).

The apparent discrepancies between the overall occupancies observed in the earlier MALDI-TOF MS and FLEXITau experiments are now superseded by the results from native MS. The fractions are now denoted as P_0_, P_m_, and P_h_ for zero, medium, and high state of phosphorylation (to distinguish the different numerical values). The common control value is P_0_-Tau from *E. coli* (Fig. 3*C*), which remains without modification, apart from N-terminal processing by removal of Met1. The medium state, reflecting normal kinase/PPase activity in cells (Fig. 3*B*), is now resolved into a series of peaks centered around a maximum at +8 P_i_ per molecule, with ∼4 sub-maxima resolved on either side (range +4 to +13 P_i_). The high state (Fig. 3*A*), reflecting normal kinase but inhibited PPase activity, appears as a series of peaks centered around a maximum at +14 P_i_, with ∼6 sub-maxima resolved on either side (range +6 to +20 P_i_).

The main conclusions are that the previous MALDI-TOF MS values agree roughly with the upper limits of the peaks resolved by native MS and that the FLEXITau values reflect the lower limits. Of note, a closer inspection of the MALDI-TOF MS results (14) shows that the experimental errors of the reported masses were up to +/−195 Da, corresponding to +/−2.5 P_i_ for individual fractions and thus +/−5 P_i_ for mass differences. On the other hand, the FLEXITau method achieved accurate occupancies of individual P-sites. However, sequence coverage was limited (75%) so that the degree of Tau phosphorylation was underestimated (Table S2 and Table S3; note that the newly covered sequences in this study contain 14 additional P-sites).

It is remarkable that Tau molecules in the many P-states are separated by 1 P_i_ unit. For a multidomain protein (or multiprotein complex) with specific functional P-sites one would expect well-defined stoichiometries of phosphorylation (by specific kinases) whereby assembly or activity are controlled; examples are ribosomes or proteasomes (44, 45). By contrast, in case of Tau all domains have a natively unfolded character, with an unusually high fraction of phosphorylatable residues (85/441 = 19%) accessible to multiple kinases and phosphatases. The degree of phosphorylation therefore appears to depend on a phosphorylation “tone” rather than on specific functional modifications. The balance between kinase and phosphatase activities depends on the experimental conditions of Tau purification, which can change rapidly. Thus, during postmortem delay, ATP is depleted (making kinases inactive) and PP2A_cat_ is activated, which makes adult human Tau appear to be in a low state of phosphorylation. Conversely, rapid isolation of brain tissue (*e.g.* with fetal Tau or brain biopsy) reveals a high state of phosphorylation. In this scheme, the high phosphorylation of AD-Tau (despite a long postmortem delay) is explained by Tau aggregation which protects them against PPases. Similarly, lowering the temperature (as in anesthesia) decreases PPase activity, with a lesser effect on kinases, causing the impression of hyperphosphorylation (46, 47, 48). Thus, although there are specific functional or diagnostic P-sites in Tau, they probably have only a small effect on the average degree of phosphorylation. By implication, the changes in phosphorylation tone would affect many cellular proteins, not just Tau, so that other proteins might also serve as diagnostic markers for AD neurodegeneration, even when they do not appear in aggregated form.

If multiple phosphorylation of a natively unfolded protein like Tau is largely a statistical property, it follows that each state of phosphorylation represents an ensemble of many Tau species, phosphorylated at different sites, depending on activity and accessibility of kinases/PPases. Certain sites may appear as reliable markers of disease, but this is not necessarily coupled to a specific function (such as aggregation or microtubule binding). Consistent with this, we find that none of the phosphorylation states led to a pronounced increase in Tau aggregation (14) even though Tau aggregation and phosphorylation seem to be coregulated during AD progression. However, the extent of phosphorylation will change some bulk properties such as charge, which could turn from net positive to net negative. This would change the interactions with other proteins (*e.g.* the cytoskeleton via microtubules and actin filaments), RNAs, or membrane surfaces and the folding of Tau from a “paperclip” to other conformations (49). In particular, phosphorylation promotes the propensity of Tau to undergo liquid-liquid phase transitions (50).

### Asymmetric distribution and hotspots of phosphorylation

**Figure 5:**
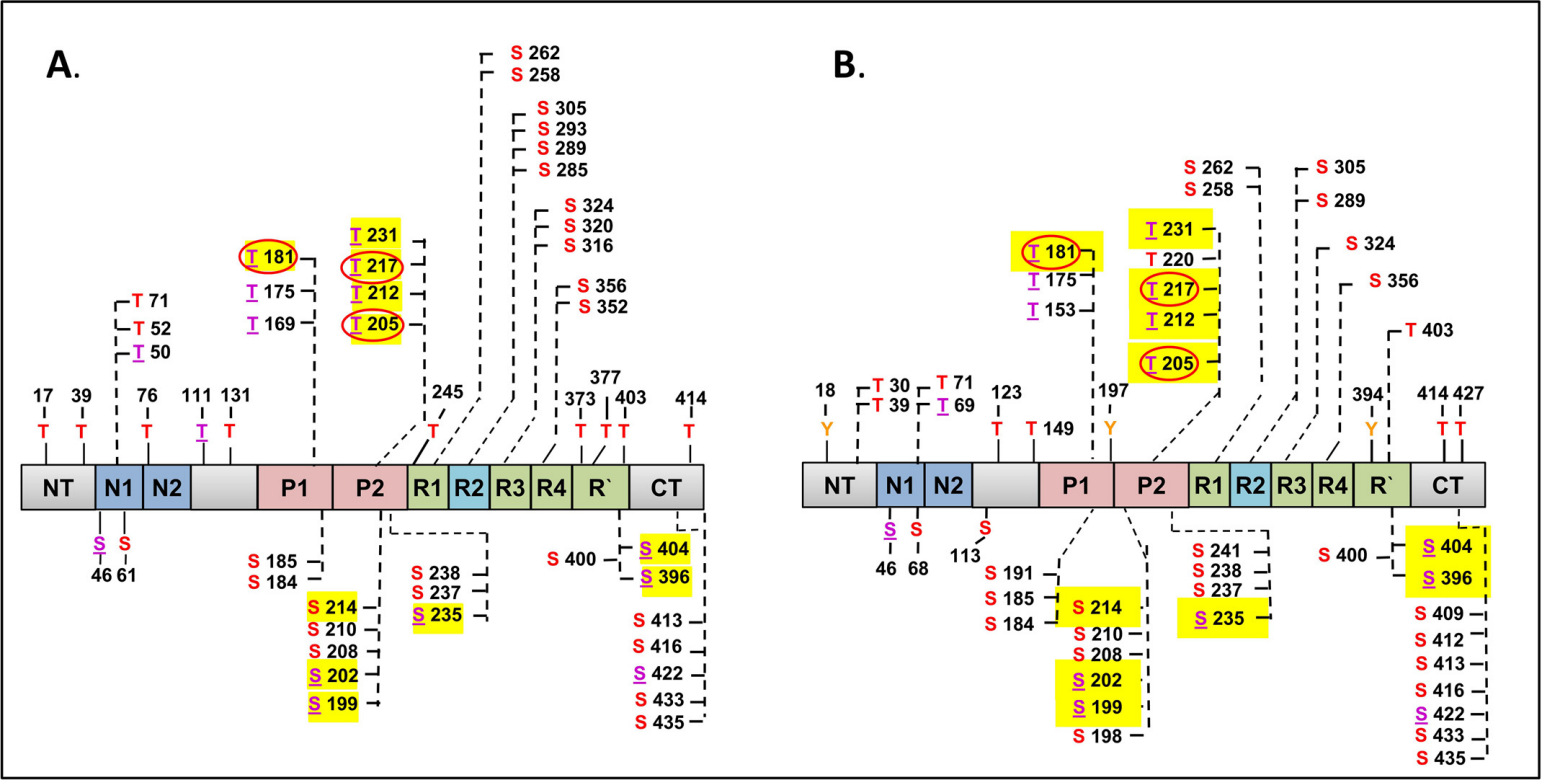
Bar diagram of full-length human Tau-2N4R protein with distribution of observed phosphorylation sites in Sf9 cells *versus* AD brain (>50 sites, mainly in C-terminal half, overlap 68%). *A*, 51 P-sites acquired by Tau in Sf9 cells (this study). *B*, 52 P-sites identified in AD-Tau; see collection of P-sites from AD brains (13). *Red*, Ser and Thr P-sites; *purple underlined*, SP or TP motifs; *yellow highlight*, high occupancy P-sites; *red circles*, P-sites important for AD diagnostics.

The sequence of Tau contains several points where phosphorylatable residues are clustered and phosphorylated, either with high occupancy at a single residue or with intermediate occupancy at two or three nearby residues. Some of these hotspots of phosphorylation are prominent as epitopes of antibodies raised against AD-Tau. The antibodies may bind to a single site within a cluster, independently of nearby P-sites (*e.g.* pT181 by antibody AT270 or pT231 by antibody AT180 (51), or require a pair of nearby phosphorylated residues (*e.g.* Ser-202+Thr-205 (AT8 (52)), Ser-212+Ser-214 (AT100 (53)), and Ser-396+Ser-404 (PHF1 (54))). These clusters are represented by the epitopes of antibodies AT270, AT8, AT100, AT180, 12E8, and PHF1, and together they could represent a substantial fraction of Tau phosphorylation (∼30% or more; Fig. 4*B* and Fig. 5 (15)). NMR studies revealed some local structural characteristics within this phosphorylated cluster region in Tau (55).

It is remarkable that all the clusters of phosphorylatable residues mentioned above are located in the C-terminal half of Tau which is responsible for MT interactions and PHF aggregation (Fig. 4). This reflects the asymmetric sensitivity of the domains toward phosphorylation. We can divide up the sequence into an N-terminal 1/3 (approximate residues 1–150, acidic character) and the C-terminal 2/3 (residues 151–441, basic character, including the proline-rich and repeat domains). The total number of 51 detected phosphorylated residues (Fig. 4) represents 51/85 = 60% of all phosphorylatable residues (Ser, Thr, and Tyr). However, the N-terminal 1/3 had only 10/27 = 37% potential sites phosphorylated, whereas in the C-terminal 2/3 the fraction of phosphorylated *versus* potential sites was almost twice as large, 41/58 = 71%. Because all Tau domains are largely unstructured and mobile in solution (56), it is unlikely that the difference in sensitivity to kinases arises from a protection of sites via folding (especially because the N-terminal part is known for its role as a “fuzzy coat” (57). Other conceivable explanations are 1) some kinases of Tau may prefer target motifs of basic character, and/or 2) kinases of Tau may first bind to acidic domains before phosphorylating other parts of the Tau chain. Direct evidence for this scenario comes from NMR studies of the kinase MARK which binds to the N-terminal region of Tau and then phosphorylates the KXGS motifs in the repeat domain (58).

### Tau phosphorylation versus aggregation in AD

The hotspots of Tau phosphorylation in Sf9 cells coincide well with the epitopes of the antibodies mentioned above, raised initially against PHFs purified from Alzheimer's brain. The coincidence of aggregated and phosphorylated states in AD tissue led to the hypothesis that (hyper-) phosphorylation predisposes Tau for aggregation and set off an extended search for kinases of Tau (33, 59). However, this effort remained inconclusive because no kinase with pronounced pro-aggregant activity was found (in contrast to FTDP17 Tau mutations, which are clearly pro-aggregant), and some phosphorylation sites were even protective against aggregation (60). The high solubility of Tau from Sf9 cells illustrates the lack of a direct causal link between phosphorylation and aggregation. In support of this, although AD-diagnostic antibodies react with Tau monomers from Sf9 cells (see (14)), this is not *per se* an indicator of cell degeneration except in diseases such as AD and transgenic mice with Tau pathology. Indirect causal links may include phosphorylation-dependent conformational changes combined with proteolytic cleavage which exposes aggregation-prone domains of Tau (*e.g.* repeat domain). Examples are cleavage by calpain or caspases (61, 62) or nonenzymatic cleavage in aging cells which causes the heterogeneous “smear” in SDS gels of AD-Tau (63). In support of this, incipient neurodegeneration correlates with the appearance of soluble tau oligomers rather than Tau-containing tangles (64, 65).

### Tau phosphorylation in cell models

As mentioned earlier, the functions of Tau in cytoskeletal remodeling, studied initially in PC12 cells and neurons (27), were well-replicated in other eukaryotic cell types of human or animal origin, including Sf9 cells (30). However, one may ask whether this also involves similar P-sites. In previous work we addressed this issue by studying CHO cells transfected with human Tau (2N4R) and a mouse neuroblastoma cell line (LAN-5) expressing endogenous Tau (42). The cells were metabolically labeled with ^32^P, followed by phosphopeptide mapping. There was a striking overlap of P-sites with the current results, especially involving the clusters of P-sites listed above. Phosphorylation became enhanced during mitosis, corresponding to the activation of the cell cycle kinase cdc2, a proline-directed kinase. Note that this phosphorylation occurred during a physiological process, without any sign of pathology or Tau aggregation, indicating that the pattern of phosphorylation serves a normal biological role. The same conclusion can be drawn from the analysis of hibernating animals where retraction and regrowth of neuronal networks are accompanied by similar changes in Tau phosphorylation (28, 66).

### Other types of PTMs in Sf9 cells

So far, we have focused on phosphorylation because it is the most conspicuous feature of Tau modification and is at the focus of biomedical research. On the other hand, research on Tau has unearthed a large variety of modifications, including chemical modifications of residues, cleavage by various proteases, or specific interactions with other regulatory components (67). For the case of Sf9 cells, the striking observation is the very low level of other modifications that have received attention in the field:

Phosphorylation of Tyr is absent or below detectability, in contrast to the abundance of Ser/Thr phosphorylation. The five Tyr residues in Tau (residues 18, 29, 197, 310, and 394) can be phosphorylated by common Tyr kinases in the brain, *e.g.* Fyn, Src, Lck, and Abl, and the proline-rich regions contain several binding sites for associated SH3 domains of the type PXXP (12, 68, 69). This makes Tau interesting as a carrier of cellular signaling. However, at least in Sf9 cells, the Tyr-dependent signaling affects at best a minute fraction of Tau.

### Acetylation of Lys

This modification is important for gene regulation but occurs in the cytoplasm as well, notably on microtubules and associated proteins (*e.g.* Lys-174 in Tau), and has been implicated in neuronal damage of aging cells (70). The low level of acetylated Tau can be explained by the high activity of histone deacetylases in Sf9 cells which makes them highly radiation-resistant (71).

Ubiquitination of Lys is a frequent Tau modification in aging or AD brains and reflects the cell's attempt to remove misfolded or aggregated Tau (59, 72). Ubiquitin binding sites have recently been revealed by high-resolution cryo-EM imaging of Tau filaments (23). However, this modification is not detectable in Sf9 cells. A simplified interpretation of these results is that Sf9 cells are “healthy” in a cellular sense: ubiquitination of Tau is very low, consistent with the absence of aggregates. Likewise, acetylation is low, as well. The phosphorylation tone is high, comparable with that of fetal neurons in absence of aggregates. Further evidence for the healthy state is that the Sf9 cells develop extensions analogous to those of differentiating neurons, stabilized by bundles of parallel microtubules and microtubule-associated Tau (30, 73). This implies that the increased Tau phosphorylation is not necessarily detrimental and occurs under both physiological and pathological conditions.

### Tau-associated proteins

In both cell types studied here (*E. coli* and Sf9), LC–MS/MS and native MS revealed traces of numerous proteins which remained associated with Tau through the purification (see Table S4, Table S5, Fig. 2, and Fig. 3). This agrees well with the multiple published interactions of Tau (74). Many of the accessory proteins belong to the class of ribonucleoprotein complexes (75, 76). Examples are proteins involved in mRNA metabolic processes, *e.g.* translation initiation and translation elongation (77). Tau expression can differentially shift both the transcriptome and the nascent proteome, and the synthesis of ribosomal proteins is reversibly dependent on Tau levels (78, 79). The presence of DNA binding proteins is reminiscent of nuclear functions of Tau in DNA protection under stress conditions (80). Finally, there are numerous examples of interactions between Tau and chaperones (81), a class that is prominent among the identified associated proteins. For example, the periplasmic chaperone protein Skp from *E. coli* interacts with membrane proteins, thus maintaining the solubility of early folding intermediates during passage through the periplasm (82). Skp is similar to Prefoldin/GimC, a cytosolic chaperone present in eukarya and archaea (83). Prefoldin, a protein chaperone used in protein folding complexes, works as a transfer protein in conjunction with a molecule of chaperonin to correctly fold other nascent proteins, such as the cytoskeletal proteins actin and tubulin.

In summary, by combining the results from native MS analysis of intact Tau and peptide identification using LC–MS/MS, we have been able to specify the state of modified Tau in eukaryotic Sf9 cells compared with the nearly unmodified state of Tau expressed in *E. coli*. Among the identified PTMs on Tau from Sf9 cells, extensive phosphorylation at Ser/Thr residues is by far the dominant modification (up to 20 P_i_ per molecule), whereas other well-known modifications are present only in minute proportions (acetylation, Tyr phosphorylation, ubiquitination). The Ser/Thr phosphorylation has a remarkably asymmetric distribution, whereby a low proportion of residues is modified in the acidic N-terminal domain of Tau, in contrast to a high proportion in the basic middle to C-terminal domains, including all of the sites detected by antibodies against Alzheimer's phospho-Tau. This indicates that a substantial degree of phosphorylation (achieved by multiple kinases) is presumably a property of normal soluble Tau in eukaryotic cells, reflecting a phosphorylation tonus dominated by net activity of kinases over phosphatases. By implication, this would be expected to affect many other proteins as well, notably natively unfolded proteins accessible to diverse kinases (84, 85). The results may help to identify further markers of disease states in neurons or elsewhere.

## Experimental procedures

### Protein preparation and purification

Tau protein (clone htau40, largest isoform in human CNS, 441 residues, Uniprot ID P10636 isoform F) expressed in *E. coli* or Sf9 cells was prepared and purified as described in (14). Expression in either system yielded Tau concentrations up to ∼230 µm without causing aggregation. In the case of Sf9 cells, the expressed Tau protein was purified from cell extracts by making use of the heat stability of Tau. Sf9 cells were incubated for 3 days at 27 °C and collected directly for preparation of phosphorylated hTau40 protein in lysis buffer (50 mm Tris-HCl, pH 7.4, 500 mm NaCl, 10% glycerol, 1% Nonidet P-40, 5 mm DTT, 10 mm EGTA, 20 mm NaF, 1 mm orthovanadate, 5 µm microcystin, and 10 µg/ml each of protease inhibitors leupeptin, aprotinin, and pepstatin) in a ratio of 1 g of Sf9 pellet to 10 ml of lysis buffer. This procedure yielded “P_m_-Tau” (alias P12-Tau in Tepper *et al.* (14) or P-Tau in Mair *et al.* (15)). To increase the phosphorylation even further, Sf9 cells were treated before harvesting for 1 h with 0.2 µm okadaic acid (a phosphatase inhibitor, Enzo Life Sciences). Next, after centrifugation, the cells were resuspended in lysis buffer and boiled in a water bath at 100 °C for 10 min. By this treatment nearly all proteins were denatured and precipitated, except for Tau, which stays soluble. The cell debris was removed by centrifuging the lysate for 15 min at 16,000 × *g.* The supernatant containing soluble Tau protein was concentrated in Millipore Amicon Ultra-4 centrifugal filter units (molecular mass cutoff of 3 kDa). This procedure yielded “P_h_-Tau” (alias P20-Tau (14) or PP-Tau (15)). The material was then applied to a size exclusion column Superdex G200 (GE Healthcare) and eluted with PBS buffer (pH 7.4; 1 mm DTT). For further experiments, the fractions containing Tau protein were pooled and concentrated again in Amicon Ultra-4 centrifugal filter units. Finally, the concentrated protein was rebuffered into 200 mm ammonium acetate, pH 7.6.

### Analysis of human Tau proteins by native MS

Purified proteins in 200 mm ammonium acetate, pH 7.6 were analyzed by nanoflow electrospray ionization MS using a Synapt HDMS (Waters and MS Vision) equipped with a 32,000 *m*/*z* range quadrupole. 3 µl of sample were introduced with an in-house manufactured gold-coated capillary needle (borosilicate thin wall with filament, outer diameter 1.0 mm, inner diameter 0.78 mm; Harvard Apparatus). The capillary voltage was set to 1.3 kV and the cone voltage to 180 V. The trap cell was filled with argon gas at a flow rate of 1 ml/min, and collisional activation was performed by applying acceleration voltages to the trap cell. The transfer cell was kept at a pressure of about 20% of that in the trap cell and a low acceleration voltage of 5 V. Spectra were calibrated externally using cesium iodide. Charge state spectra were deconvoluted by the program UniDec version 2.7.3 (86).

### Identification of peptide sequences and posttranslational modifications by LC–MS/MS

In-solution tryptic digestion of proteins was performed as described previously (87). In brief, Tau protein samples were subjected to reduction of cysteine residues with 5 mm Tris(2-carboxyethyl)phosphine and alkylation of free thiol groups with 50 mm iodoacetamide/50 mm NH_4_HCO_3_ and digested with trypsin overnight at 37 °C. Peptide mixtures were analyzed by nano-HPLC–electrospray ionization–MS/MS using a Q Exactive instrument directly coupled to an UltiMate 3000 RSLCnano HPLC system (both Thermo Fisher Scientific, Dreieich, Germany) equipped with a Nanospray Flex ion source with DirectJunction (Thermo Fisher Scientific). Peptides were washed and preconcentrated on PepMap^TM^ C18 precolumns (5 mm × 300 µm inner diameter; Thermo Fisher Scientific) and separated using AcclaimPepMap^TM^ RSLC columns (50 cm × 75 µm inner diameter; pore size 100 Å; particle size 2 µm) at a flow rate of 250 nl/min and 40–43 °C. For peptide elution, binary solvent systems were used consisting of 0.1% (v/v) formic acid (solvent A) and 0.1% (v/v) formic acid/86% (v/v) acetonitrile (solvent B) with a gradient of 4–42% solvent B in 50 min, 42–95% in 5 min, and 5 min at 95%. The Q Exactive was operated with the following parameters: MS survey scans ranging from *m*/*z* 375–1,700 at a resolution of 70,000 (at *m*/*z* 200), an automatic gain control of 3 × 10^6^ ions, and a maximum injection time of 60 ms. A TOP12 method was used for higher energy collisional dissociation of precursor peptides (*z* ≥ 2) in the orbitrap applying a normalized collision energy of 28%, an automatic gain control of 1 × 10^5^ ions, and maximum injection time of 120 ms. The dynamic exclusion time for previously fragmented precursors was set to 45 s.

Data were analyzed using Maxquant software version 1.5.5.1 (88) searching against the sequence of human Tau-F (Uniprot ID P10636-8) and UniProt organism-specific sequence databases for *E. coli* and *Spodoptera frugiperda* (version 2018_08, 4,344 and 26,434 entries, respectively) allowing a maximum of four missed cleavages of trypsin and a mass tolerance of 4.5 ppm for precursor and 20 ppm for fragment ions. Methionine oxidation, acetylation of lysine and protein N terminus, and phosphorylation at serine, threonine, and tyrosine residues were specified as variable modifications, and carbamidomethylation of cysteine was specified as a fixed modification. Additional searches were performed with an extended range of variable modifications including ubiquitination (diglycine at lysine) and *O*-GlcNAc modification of serine and threonine. However, these modifications were observed only sporadically and in low intensity and borderline significance and were therefore excluded from the final search. The lists of both peptide and protein identifications were filtered applying a threshold for the false discovery rate of <0.01.

## Data availability

MS raw data and result files have been deposited to the ProteomeXchange Consortium via the PRIDE repository (89) and are publicly accessible from its website with the data set identifier PXD020985.
