# Supplementary material for: A combinatorial native MS and LC-MS/MS approach reveals high intrinsic phosphorylation of human Tau but minimal levels of other key modifications
Source: J Biol Chem. 2021 Jan 13;295(52):18213–25. doi: 10.1074/jbc.RA120.015882 (PMC7939451; doi:10.1074/jbc.RA120.015882)
Supplement: Supplementary file 1 [file mmc1.zip › 163274_2_supp_618584_qjsyqq.docx]

**SUPPORTING INFORMATION APPENDIX**

**A combinatorial native MS and LC-MS/MS approach reveals high intrinsic phosphorylation of human tau but minimal levels of other key modifications**

Authors:

Friedel Drepper^1,2,¶^, Jacek Biernat^3,¶^, Senthillvelrajan Kaniyappan^3,4^, Helmut E. Meyer^5,6^,

Eva Maria Mandelkow^3,7^, Bettina Warscheid^1,2*^, Eckhard Mandelkow^3,7*^

^1^ Biochemistry and Functional Proteomics, Institute of Biology II, University of Freiburg, Schänzlestr. 1, 79104 Freiburg, Germany

^2^ Signalling Research Centres BIOSS and CIBSS, University of Freiburg

^3^ DZNE (German Center for Neurodegenerative Diseases), Venusberg-Campus 1, Building 99, 53127 Bonn, Germany

^4^ Department of Neurodegenerative Diseases and Geriatric Psychiatry, University of Bonn, Venusberg-Campus 1, Building 82, 53127 Bonn, Germany.

^5^ Medical Proteome -Center, Ruhr-University Bochum, Universitätsstraße 150, 44801 Bochum, Germany

^6^ Leibniz-Institute für Analytical Sciences (ISAS), Biomedical Research Dortmund, Germany

^7^ CAESAR Research Center, Ludwig-Erhard-Allee 2, 53127 Bonn, Germany

* Corresponding authors:

Eckhard Mandelkow, E-mail: [mand@mpasmb.desy.de](mailto:mandelkow@mpasmb.desy.de)

Bettina Warscheid, E-Mail: bettina.warscheid@biologie.uni-freiburg.de

¶ Friedel Drepper, Jacek Biernat are joint first authors

**Running title: Native MS reveals high intrinsic phosphorylation of Tau**

**LIST OF SUPPORTING INFORMATION MATERIAL**

- **Table S1: A)** Phosphorylation sites on Tau protein identified by LC-MS/MS.
  - (**Tables S1B-D** are provided in a separate xlsx format document)
- **Table S2:** P-sites in human Tau (2N4R) expressed in Sf9 cells by three independent studies
- **Table S3:** Sequence coverage of Tau peptides
- **Table S4:** Ten most abundant Tau-associated proteins identified by LC-MS/MS analysis in samples of purified Po-Tau expressed in *E. coli*.
- **Table S5: A)** 20 most abundant proteins co-purifying with Tau identified by LC-MS/MS in fractions of P_m_-Tau and P_h_-Tau from Sf9 cells**.**
- **Table S5: B)** Known functions of proteins co-purified with Tau.

**Table S1: Phosphorylation sites on Tau protein identified by LC-MS/MS.** Note that phosphorylation is observed only on Ser or Thr, not on Tyr. Tau proteins (fractions P_0_-Tau, P_m_-Tau, P_h_-Tau and neuronal Tau^A152T^) were proteolytically digested with trypsin. Peptide mixtures were analyzed by LC-MS on a Q Exactive mass spectrometer using HCD for peptide fractionation. MS raw data were analyzed using MaxQuant/Andromeda (version 1.5.5.1). Data presented in this table were extracted from the MaxQuant output files "evidence.txt". **(A)** Summary of phosphosites identified with a PEP value of < 0.01 and a localization probability of > 0.75. **(B)** P_m_-Tau and P_h_-Tau phosphopeptides identified with a PEP value of < 0.01 are listed with selected features in this spreadsheet. **(C)** All P_0_-Tau, P_m_-Tau and P_h_-Tau peptides identified in this analysis. **(D)** Phosphorylated and unphosphorylated peptides identified in neuronal Tau purified from mouse brain using the Tau^A152T^ specific antibody 1C5 (MB_1C5).

Datasets **B-D** are provided in a separate file in xlsx format.

**Table S1A**

| Peptide  No. | Residues | P-Site | Antibody  against  P-epitope | Peptide sequences |
| --- | --- | --- | --- | --- |
| 1 | 6-24 | T17 |  | QEFEVMEDHAG**T**YGLGDRK |
| 2 | 25-44 | T39 |  | DQGGYTMHQDQEGD**T**DAGLK |
| 3 | 24-67 | S46 |  | KDQGGYTMHQDQEGDTDAGLKE**S**PLQTPTEDGSEEPGSETSDAK |
| 4 | 24-67 | T50 |  | KDQGGYTMHQDQEGDTDAGLKESPLQ**T**PTEDGSEEPGSETSDAK |
| 5 | 45-67 | T52 |  | ESPLQTP**T**EDGSEEPGSETSDAK |
| 6 | 45-67 | S61 |  | ESPLQTPTEDGSEEPG**S**ETSDAK |
| 7 | 68-87 | T71 |  | STP**T**AEDVTAPLVDEGAPGK |
| 8 | 68-87 | T76 |  | STPTAEDV**T**APLVDEGAPGK |
| 9 | 88-126 | T111 |  | QAAAQPHTEIPEGTTAEEAGIGD**T**PSLEDEAAGHVTQAR |
| 10 | 127-143 | S131 |  | MVSK**S**KDGTGSDDKKAK |
| 11 | 164-174 | T169 |  | GQANA**T**RIPAK |
| 12 | 171-194 | T175 |  | IPAK**T**PPAPKTPPSSGEPPKSGDR |
| 13 | 175-194 | T181 | AT270 | TPPAPK**T**PPSSGEPPKSGDR |
| 14 | 175-194 | S184 |  | TPPAPKTPP**S**SGEPPKSGDR |
| 15 | 175-194 | S185 |  | TPPAPKTPPS**S**GEPPKSGDR |
| 16 | 195-209 | S199 |  | SGYS**S**PGSPGTPGSR |
| 17 | 195-209 | S202 | AT8 | SGYSSPG**S**PGTPGSR |
| 18 | 195-221 | T205 | AT8 | SGYSSPGSPG**T**PGSRSRTPSLPTPPTR |
| 19 | 195-221 | S208 |  | SGYSSPGSPGTPG**S**RSRTPSLPTPPTR |
| 20 | 210-224 | S210 |  | **S**RTPSLPTPPTREPK |
| 21 | 210-221 | T212 | AT100 | SR**T**PSLPTPPTR |
| 22 | 212-221 | S214 | AT100 | TP**S**LPTPPTR |
| 23 | 212-224 | T217 |  | TPSLP**T**PPTREPK |
| 24 | 226-240 | T231 | AT180 | VAVVR**T**PPKSPSSAK |
| 25 | 226-242 | S235 |  | VAVVRTPPK**S**PSSAKSR |
| 26 | 225-240 | S237 |  | KVAVVRTPPKSP**S**SAK |
| 27 | 225-242 | S238 |  | KVAVVRTPPKSPS**S**AKSR |
| 28 | 243-254 | T245 |  | LQ**T**APVPMPDLK |
| 29 | 255-267 | S258 |  | NVK**S**KIGSTENLK |
| 30 | 260-267 | S262 | 12E8 | IG**S**TENLK |
| 31 | 281-290 | S285 |  | KLDL**S**NVQSK |
| 32 | 281-290 | S289 |  | KLDLSNVQ**S**K |
| 33 | 291-317 | S293 |  | CG**S**KDNIKHVPGGGSVQIVYKPVDLSK |
| 34 | 299-317 | S305 |  | HVPGGG**S**VQIVYKPVDLSK |
| 35 | 299-321 | S316 |  | HVPGGGSVQIVYKPVDL**S**KVTSK |
| 36 | 318-340 | S320 |  | VT**S**KCGSLGNIHHKPGGGQVEVK |
| 37 | 322-343 | S324 |  | CG**S**LGNIHHKPGGGQVEVKSEK |
| 38 | 350-370 | S352 |  | VQ**S**KIGSLDNITHVPGGGNKK |
| 39 | 354-370 | S356 | 12E8 | IG**S**LDNITHVPGGGNKK |
| 40 | 370-379 | T373 |  | KIE**T**HKLTFR |
| 41 | 370-383 | T377 |  | KIETHKL**T**FRENAK |
| 42 | 384-406 | S396 | PHF1 | AKTDHGAEIVYK**S**PVVSGDTSPR |
| 43 | 386-406 | S400 |  | TDHGAEIVYKSPVV**S**GDTSPR |
| 44 | 384-406 | T403 |  | AKTDHGAEIVYKSPVVSGD**T**SPR |
| 45 | 396-406 | S404 | PHF1 | SPVVSGDT**S**PR |
| 46 | 407-438 | S413 |  | HLSNVS**S**TGSIDMVDSPQLATLADEVSASLAK |
| 47 | 407-438 | T414 |  | HLSNVSS**T**GSIDMVDSPQLATLADEVSASLAK |
| 48 | 407-438 | S416 |  | HLSNVSSTG**S**IDMVDSPQLATLADEVSASLAK |
| 49 | 407-438 | S422 |  | HLSNVSSTGSIDMVD**S**PQLATLADEVSASLAK |
| 50 | 407-438 | S433 |  | HLSNVSSTGSIDMVDSPQLATLADEV**S**ASLAK |
| 51 | 407-438 | S435 |  | HLSNVSSTGSIDMVDSPQLATLADEVSA**S**LAK |

**Table S2: P-sites in human Tau (2N4R) expressed in Sf9 cells by three independent studies: Tepper et al. (2014) [MALDI-TOF MS], Mair et al. (2016) [FLEXITau] and current work [native MS and LC-MS/MS].** Column 1, potential P-sites in full-length Tau (residues S, T, Y); columns 2-4, observed P-sites in Tau from Sf9 cells; column 5, P-sites in Tau from human AD brain (Hanger, 2020). Additional sites observed in the current work are marked in red, sites observed in previous but not in present study in blue.

| Potential Site | P-Sites obs. | P-Sites obs. | P-Sites obs. | P-Sites reported |
| --- | --- | --- | --- | --- |
| S,T, Y in htau 2N4R | Tepper 2014 | Mair 2016 | This work | AD brain  (Hanger, 2020) |
| T 17 |  |  | T 17 |  |
| Y 18 |  |  |  | Y 18 |
| T 30 |  |  |  | T 30 |
| T 39 |  |  | T 39 | T 39 |
| S 46 |  |  | S 46 | S 46 |
| T 50 |  |  | T 50 |  |
| T 52 |  |  | T 52 |  |
| S 61 |  |  | S 61 |  |
| S 68 | S 68 | S 68 |  | S 68 |
| T 71 |  |  |  |  |
| T 69 | T 69 | T 69 |  | T 69 |
| T 71 | T 71 |  | T 71 | T 71 |
| T 76 | T 76 | T 76 | T 76 |  |
| T 111 |  |  | T 111 |  |
| S 113 |  |  |  | S 113 |
| T 123 |  |  |  | T 123 |
| S 131 | S 131 |  | S 131 |  |
| T 135 | T 135 |  |  |  |
| T 149 | T 149 |  |  | T 149 |
| T 153 | T 153 | T 153 |  | T 153 |
| T 169 |  |  | T 169 |  |
| T 175 |  | T 175 | T 175 | T 175 |
| T 181 |  | T 181 | T 181 | T 181 |
| S 184 |  | S 184 | S 184 | S 184 |
| S 185 |  |  | S 185 | S 185 |
| S 191 |  |  |  | S 191 |
| Y 197 |  |  |  | Y 197 |
| S 198 |  |  |  | S 198 |
| S 199 |  | S 199 | S 199 | S 199 |
| S 202 | S 202 | S 202 | S 202 | S 202 |
| T 205 | T 205 | T 205 | T 205 | T 205 |
| S 208 |  |  | S 208 | S 208 |
| S 210 |  | S 210 | S210 | S 210 |
| T 212 | T 212 | T 212 | T 212 | T 212 |
| S 214 | S 214 | S 214 | S 214 | S 214 |
| T 217 |  | T 217 | T 217 | T 217 |
| T 220 |  |  |  | T 220 |
| T 231 | T 231 | T 231 | T 231 | T 231 |
| S 235 | S 235 | S 235 | S 235 | S 235 |
| S 237 |  |  | S 237 | S 237 |
| S 238 |  |  | S 238 | S 238 |
| S 241 |  |  |  | S 241 |
| T 245 |  |  | T 245 |  |
| S 258 |  |  | S 258 | S 258 |
| S 262 | S 262 | S 262 | S 262 | S 262 |
| S 285 |  |  | S 285 |  |
| S 289 |  |  | S 289 | S 289 |
| S 293 |  | S 293 | S 293 |  |
| S 305 | S 305 |  | S 305 | S 305 |
| Y 310 | Y 310 |  |  |  |
| S 316 | S 316 |  | S 316 |  |
| S 320 |  |  | S 320 |  |
| S 324 |  | S 324 | S 324 | S 324 |
| S 352 |  |  | S 352 |  |
| S 356 | S 356 | S 356 | S 356 | S 356 |
| T 373 |  |  | T 373 |  |
| T 377 |  |  | T 377 |  |
| Y 394 |  |  |  | Y 394 |
| S 396 | S 396 | S 396 | S 396 | S 396 |
| S 400 |  |  | S 400 | S 400 |
| T 403 |  |  | T 403 | T 403 |
| S 404 | S 404 | S 404 | S 404 | S 404 |
| S 409 |  |  |  | S 409 |
| S 412 |  |  |  | S 412 |
| S 413 |  |  | S 413 | S 413 |
| T 414 |  |  | T 414 | T 414 |
| S 416 |  |  | S 416 | S 416 |
| S 422 |  |  | S 422 | S 422 |
| T 427 |  |  |  | T 427 |
| S 433 |  |  | S 433 | S 433 |
| S 435 |  |  | S 435 | S 435 |

**Table S3: Sequence coverage of Tau peptides in Mair et al., 2016 (M) and current work (D) with all P-sites detected**.

Red letters= P-sites observed in current work. Blue=sites observed in Mair et al., 2016 study.

Note that the newly covered sequences in this study (symbol ***) contain 14 observed phosphorylation sites (red letters S, T). Sequences covered previously but not in current study (symbol +++) contain only 1 observed phosphorylation site (blue, T153).

Thus, the excess of new sequence coverage is sufficient to explain the higher occupancy in the current study.

grey: missing residues in sequence coverage.

*** Gaps in (M)

+++ Gaps in (D);

--- Gaps in both.

| **M 1 MAEPRQEFEVMEDHAGTYGLGDRKDQGGYTMHQDQEGDTDAGLK** |
| --- |
| **D MAEPRQEFEVMEDHAGTYGLGDRKDQGGYTMHQDQEGDTDAGLK** |
| ----- |
| **M 45 ESPLQTPTEDGSEEPGSETSDAKSTPTAE** |
| **D ESPLQTPTEDGSEEPGSETSDAKSTPTAE** |
|  |
| **M 74 DVTAPLVDEGAPGKQAAAQPHTEIPEGTT** |
| **D DVTAPLVDEGAPGKQAAAQPHTEIPEGTT** |
|  |
| **M 103 AEEAGIGDTPSLEDEAAGHVTQARMVSKSKDGTGSDDKKAKGADGKTK** |
| **D AEEAGIGDTPSLEDEAAGHVTQARMVSKSKDGTGSDDKKAKGADGKTK** |
| *****************------- |
| **M 151 IATPRGAAPPGQKGQANATRIPAKTPPAPKTPPSSGEPPKSGDRSGY** |
| **D IATPRGAAPPGQKGQANATRIPAKTPPAPKTPPSSGEPPKSGDRSGY** |
| +++++++++++++ **** |
| **M 198 SSPGSPGTPGSRSRTPSLPTPPTREPKKVAVVRTPPKSPSSAKSRL** |
| **D SSPGSPGTPGSRSRTPSLPTPPTREPKKVAVVRTPPKSPSSAKSRL** |
| ********* |
| **M 244 QTAPVPMPDLKNVKSKIGSTENLKHQPGGGK** |
| **D QTAPVPMPDLKNVKSKIGSTENLKHQPGGGK** |
| ***** ------- |
| **M 275 VQIINKKLDLSNVQSKCGSKDNIKHVPGGGS** |
| **D VQIINKKLDLSNVQSKCGSKDNIKHVPGGGS** |
| **++++++ ********** |
| **M 306 VQIVYKPVDLSKVTSKCGSLGNIHHKPGGGQ** |
| **D VQIVYKPVDLSKVTSKCGSLGNIHHKPGGGQ** |
| ******** |
| **M 337 VEVKSEKLDFKDRVQSKIGSLDNITHVPGGGN** |
| **D VEVKSEKLDFKDRVQSKIGSLDNITHVPGGGN** |
| **+++---****** |
| **M 369 KKIETHKLTFRENAKAKTDHGAEIVYKSPVVS** |
| **D KKIETHKLTFRENAKAKTDHGAEIVYKSPVVS** |
| ********* |
| **M 401 GDTSPRHLSNVSSTGSIDMVDSPQLATLADEVSASLAKQGL** |
| **D GDTSPRHLSNVSSTGSIDMVDSPQLATLADEVSASLAKQGL** |
| **********************************---** |

**Table S4:** **Ten most abundant Tau-associated proteins identified by LC-MS/MS analysis in samples of purified P_o_-Tau expressed in *E. coli*.** Protein samples were digested with trypsin followed by LC-MS/MS. Data were analyzed using MaxQuant software searching against the sequence of human Tau-F (Uniprot Id P10636-8) and Uniprot organism specific databases *E. coli* and *Spodoptera frugiperda* (SPOFR). iBAQ = intensity-based absolute quantification correcting for differences in the number of predicted peptides per protein. Only the 10 most abundant proteins per sample are listed. Average of two biological replicates. Note the frequency of RNA- or DNA-binding proteins copurified with Tau.

| **UniProt Id** | **Source** | **Gene name** | **Protein name** | **Pept. count** | **Seq.**  **cov.**  **[%]** | **Mol. wt [kDa]** | **iBAQ intensity/E+10**  **ctrl-Tau** | **rank ctrl-Tau** |
| --- | --- | --- | --- | --- | --- | --- | --- | --- |
| P10636-8 | HUMAN | MAPT | **Tau** | 102 | 95.9 | 45.849 | 3.10 | 1 |
| P0A7N9 | ECOLI | rpmG | **50S ribosomal protein L33** | 8 | 80 | 6.3715 | 0.62 | 2 |
| P0AEU7 | ECOLI | skp | **Chaperone protein Skp** | 16 | 76.4 | 17.688 | 0.35 | 3 |
| P0A7M9 | ECOLI | rpmE | **50S ribosomal protein L31** | 8 | 90 | 7.871 | 0.20 | 4 |
| P0ADN2 | ECOLI | yifE | **UPF0438 protein YifE** | 21 | 87.5 | 13.133 | 0.19 | 5 |
| P0ACF0 | ECOLI | hupA | **DNA-binding protein HU-alpha** | 10 | 71.1 | 9.5349 | 0.14 | 6 |
| P60624 | ECOLI | rplX | **50S ribosomal protein L24** | 17 | 79.8 | 11.316 | 0.10 | 7 |
| P0ADK8 | ECOLI | yibL | **Uncharacterized protein** | 10 | 53.3 | 13.696 | 0.04 | 8 |
| P0ACF4 | ECOLI | hupB | **DNA-binding protein HU-β** | 8 | 68.9 | 9.2255 | 0.04 | 9 |
| P68191 | ECOLI | sra | **ribosome-associated protein** | 4 | 57.8 | 5.0958 | 0.04 | 10 |

**Table S5A: 20 most abundant proteins co-purifying with Tau identified by LC-MS/MS in fractions of P_m_-Tau and P_h_-Tau from Sf9 cells.** For protein functions see Table S5B.

| **UniProt Id** | **Source** | **Gene name** | **Peptide count** | **Seq. cov. [%]** | **Mol.**  **mass**  **[kDa]** | **iBAQ**  **P_m_-Tau** | **iBAQ P_h_-Tau** | **rank P_m_**  **Tau** | | **rank P_h_**  **Tau** |
| --- | --- | --- | --- | --- | --- | --- | --- | --- | --- | --- |
| P10636-8 | HUMAN | MAPT | 102 | 95.9 | 45.849 | 1.8E+10 | 2.6E+10 | | 1 | 1 |
| A0A2H1VYE4 | SPOFR | SFRICE_014287 | 4 | 20.0 | 12.545 | 4.1E+09 | 1.1E+09 | | 2 | 5 |
| Q8WQJ0 | SPOFR | SFRICE_016047 | 20 | 100 | 11.525 | 1.8E+09 | 1.2E+09 | | 4 | 2 |
| A0A1C7D1B9 | SPOFR | CALM2 | 19 | 99.3 | 16.810 | 1.8E+09 | 1.1E+09 | | 3 | 3 |
| P62925 | SPOFR | eIF-5A | 22 | 86.9 | 17.525 | 1.2E+09 | 1.1E+09 | | 5 | 4 |
| A0A2H1W3F3 | SPOFR | SFRICE_023497 | 14 | 48.8 | 22.506 | 1.1E+09 | 7.7E+08 | | 6 | 6 |
| A0A2H1W368 | SPOFR | SFRICE_003581 | 27 | 80.4 | 19.592 | 9.0E+08 | 6.9E+08 | | 7 | 7 |
| A0A2H1W7P4 | SPOFR | SFRICE010198.2 | 14 | 83.5 | 11.217 | 7.5E+08 | 4.3E+08 | | 9 | 13 |
| I7F433 | SPOFR | CFL | 22 | 92.6 | 16.995 | 5.4E+08 | 5.3E+08 | | 12 | 9 |
| Q962R7 | SPOFR | RpS12 | 14 | 84.2 | 14.892 | 3.6E+08 | 6.5E+08 | | 18 | 8 |
| A0A2H1WD76 | SPOFR | SFRICE_001903 | 35 | 64.5 | 43.714 | 5.6E+08 | 4.5E+08 | | 11 | 12 |
| A0A2H1VL41 | SPOFR | SFRICE_006583 | 9 | 59.2 | 13.341 | 5.3E+08 | 4.7E+08 | | 13 | 10 |
| A0A2H1VF43 | SPOFR | SFRICE_021632 | 14 | 56.8 | 12.194 | 7.6E+08 | 2.4E+08 | | 8 | 26 |
| A0A2H1W542 | SPOFR | SFRICE_011912 | 8 | 46.9 | 6.968 | 5.0E+08 | 4.2E+08 | | 14 | 14 |
| A0A2H1VA11 | SPOFR | SFRICE_005530 | 9 | 73.9 | 16.921 | 5.9E+08 | 3.2E+08 | | 10 | 17 |
| A0A2H1WWX 88 | SPOFR | SFRICE_031205 | 8 | 78.7 | 10.157 | 4.7E+08 | 4.0E+08 | | 15 | 16 |
| A0A2H1W5E9 | SPOFR | SFRICE_008465 | 10 | 82.7 | 12.446 | 3.7E+08 | 4.1E+08 | | 16 | 15 |
| A0A2H1WQB1 | SPOFR | SFRICE_004596 | 13 | 90.0 | 10.196 | 2.4E+08 | 4.7E+08 | | 27 | 11 |
| A0A2H1V4G9 | SPOFR | SFRICE_023010 | 12 | 38.3 | 21.015 | 3.7E+08 | 2.9E+08 | | 17 | 19 |
| A0A2H1VTT2 | SPOFR | SFRICE_010125 | 6 | 62.1 | 14.986 | 2.8E+08 | 3.0E+08 | | 23 | 18 |
| A0A2H1WKM222 | SPOFR | SFRICE_038968 | 2 | 64.9 | 6.6093 | 3.1E+08 | 2.5E+08 | | 20 | 24 |
| A0A2H1VSI0 | SPOFR | SFRICE_022351 | 15 | 23.6 | 46.453 | 3.6E+08 | 1.9E+08 | | 19 | 28 |
| Q962Q2 | SPOFR | RpS28 | 8 | 61.5 | 7.3184 | 1.0E+08 | 2.8E+08 | | 75 | 20 |

**Table S5B: Known functions of proteins co-purified with Tau.**

|  |
| --- |
| \| **Protein ID** **Source Gene name** **Function** \| \| --- \|  \| A0A2H1VYE4 \| SPOFR \| SFRICE_014287 \| uncharacterized protein \| \| --- \| --- \| --- \| --- \| \| Q8WQJ0 \| SPOFR \| SFRICE_016047 \| 60S acidic ribosomal protein P2; structural constituent  of ribosome; translational elongation \| \| A0A1C7D1B9 \| SPOFR \| CALM2 \| calcium ion binding \| \| P62925 \| SPOFR \| eIF-5A \| ribosome binding; translation elongation factor activity \| \| A0A2H1W3F3 \| SPOFR \| SFRICE_023497 \| uncharacterized protein \| \| A0A2H1W368 \| SPOFR \| SFRICE_003581 \| transcription factor BTF3 (protein inferred by homology) \| \| A0A2H1W7P4 \| SPOFR \| SFRICE 010198.2 \| ATP binding prot (chaperon) \| \| I7F433 \| SPOFR \| CFL \| cofilin; actin binding protein \| \| Q962R7 \| SPOFR \| RpS12 \| 40S ribosomal protein S12 \| \| A0A2H1WD76 \| SPOFR \| SFRICE_001903 \| RNA binding; protein predicted; unreviewed \| \| A0A2H1VL41 \| SPOFR \| SFRICE_006583 \| DNA binding protein; component of nucleus (predicted) \| \| A0A2H1VF43 \| SPOFR \| SFRICE_021632 \| uncharacterized protein \| \| A0A2H1W542 \| SPOFR \| SFRICE_011912 \| translation machinery associated protein 7 \| \| A0A2H1VA11 \| SPOFR \| SFRICE_005530 \| proton-transporting ATP synthase activity;  ATP synthesis proton coupled transport \| \| A0A2H1WWX8 \| SPOFR \| SFRICE_031205 \| chaperon mediated protein transport \| \| A0A2H1W5E9 \| SPOFR \| SFRICE_008465 \| protein N-linked glycosylation, protein in ER in  oligosaccharyl transferase complex; integral  component of membrane \| \| A0A2H1WQB1 \| SPOFR \| SFRICE_004596 \| DNA binding; protein predicted; unreviewed \| \| A0A2H1V4G9 \| SPOFR \| SFRICE_023010 \|  \| \| A0A2H1VTT2 \| SPOFR \| SFRICE_010125 \| heme binding; metal ion binding \| \| A0A2H1WKM2 \| SPOFR \| SFRICE_038968 \| translation initiation factor activity \| \| A0A2H1VSI0 \| SPOFR \| SFRICE_022351 \| poly-ADP-D ribose binding protein; ubiquitin protein ligase;  zinc ion binding \| \| Q962Q2 \| SPOFR \| RpS28 \| 40S ribosomal protein S28 \| |
